# Meter enhances the subcortical processing of speech sounds at a strong beat

**DOI:** 10.1038/s41598-020-72714-z

**Published:** 2020-09-29

**Authors:** Il Joon Moon, Soojin Kang, Nelli Boichenko, Sung Hwa Hong, Kyung Myun Lee

**Affiliations:** 1 Department of Otorhinolaryngology-Head and Neck Surgery, Samsung Medical Center, Sungkyunkwan University School of Medicine, Seoul, South Korea; 2 Hearing Research Laboratory, Samsung Medical Center, Seoul, South Korea; 3 Department of Physics, Chungnam National University, Daejeon, South Korea; 4 Department of Otorhinolaryngology-Head and Neck Surgery, Samsung Changwon Hospital, Sungkyunkwan University School of Medicine, Changwon, South Korea; 5 Music and Brain Research Lab and School of Humanities and Social Sciences, Korea Advanced Institute of Science and Technology, Youseong Daehakro 291, Daejeon, 34141 South Korea; 6 Graduate School of Culture Technology, Korea Advanced Institute of Science and Technology, Daejeon, South Korea

**Keywords:** Auditory system, Sensory processing

## Abstract

The temporal structure of sound such as in music and speech increases the efficiency of auditory processing by providing listeners with a predictable context. Musical meter is a good example of a sound structure that is temporally organized in a hierarchical manner, with recent studies showing that meter optimizes neural processing, particularly for sounds located at a higher metrical position or strong beat. Whereas enhanced cortical auditory processing at times of high metric strength has been studied, there is to date no direct evidence showing metrical modulation of subcortical processing. In this work, we examined the effect of meter on the subcortical encoding of sounds by measuring human auditory frequency-following responses to speech presented at four different metrical positions. Results show that neural encoding of the fundamental frequency of the vowel was enhanced at the strong beat, and also that the neural consistency of the vowel was the highest at the strong beat. When comparing musicians to non-musicians, musicians were found, at the strong beat, to selectively enhance the behaviorally relevant component of the speech sound, namely the formant frequency of the transient part. Our findings indicate that the meter of sound influences subcortical processing, and this metrical modulation differs depending on musical expertise.

## Introduction

Formed from regularity, the temporal structure of sound leads listeners to efficiently process auditory information. Regular accents in speech or the emphasis on the first beat in a 3/4 time waltz are good examples of this temporal structure. Particularly, the temporal structure of music is organized into equally-spaced beats, with the grouping of regular beats constructing the meter. In the hierarchical nature of meter, beats that are relatively stronger are considered to be on higher metrical levels. For example, in a quadruple meter like 4/4 time, a series of isochronous beats are heard as a repeated cycle of four beats—strong, weak, medium, and weak. In this case, the first and third beats have the highest and second-highest metrical positions, respectively, whereas the second and fourth beats have the lowest. Humans construct meter by grouping beats, and then use the hierarchical structure of meter to predict incoming sounds. While beat extraction is possible for a select group of animals such as parrots and sea lions, no evidence that animals can perceive true musical meter has yet been found^1^.

It has been suggested that meter guides real-time attention during listening^2, 3^. More attention is allocated to the metrically higher positions, leading to a heightened sensitivity to events at beats having higher metrical levels^2, 3^. This has been evidenced by a wealth of behavioral research; for example, auditory tasks such as pitch judgements and distinguishing just-noticeable temporal differences are performed better at higher metrical positions^4–6^. Even visual tasks, such as letter identification and word recognition, show better performance when stimuli are provided at higher metrical levels^7, 8^. Neurophysiological studies also showed that sounds on metrically strong beats are differently processed in the brain. Specifically, evoked potentials, such as N1, P2, and mismatch negativity (MMN), were also found to be higher or earlier when the oddball was provided at metrically higher positions^9–13^. Taken together, the metrical structure of sounds is known to enhance behavioral performance and the neural processing of the auditory pathway in the human brain.

To date, though, no direct evidence has been found showing the effect of the metrical hierarchy of sounds on processing in the human brainstem, which is the subcortical structure that connects the auditory periphery and the cortex. Recent studies have revealed that the brainstem is sensitive to the context of auditory information. The probability effect of stimuli has been found at the brainstem level; speech and musical sounds are more accurately encoded when they are presented at higher rather than lower probabilities^14–18^, and conversely, when speech sounds are provided as deviant stimuli, the amplitudes of brainstem responses are reduced^19^. Given the brainstem sensitivity to context, the metrical structure of sounds should also modulate subcortical processing. To examine this effect, this study measures brainstem responses to a sound presented at four different metrical positions. To provide metrical hierarchy, we overlay a speech sound, /da/, with a repeating series of four tones, in which the first tone has a higher pitch than the following three tones, making the first tone the strong beat with the highest metrical position. Via electrodes on the scalp, we measure far-field auditory brainstem responses to the sound at the different metrical positions. We mainly analyze the frequency-following response (FFR), which reflects the tonic component of the response generated by the phase-locking of neuronal ensembles mainly in the auditory brainstem and midbrain^20^. It is expected that the sound presented at the highest metrical position, that is, /da/ at every first beat, will be enhanced. Enhanced processing of strong beats in the brainstem could be related to the stability and/or fidelity of neural responses; therefore, we analyzed both FFR consistency for response stability and the FFR spectrum for response fidelity. Given that prior works have shown the context effect as indexed by heightened fundamental (F0) and first formant (F1) frequencies^14, 16, 17^, our spectral analysis focused on F0 and F1.

In addition, this study investigated how the effect of metrical hierarchy on subcortical encoding differs between musicians and non-musicians. Prior works observed that musicians have a strong representation of meter^21^ and perceive metrical structure better than non-musicians^22^. Electrophysiological studies have also demonstrated that the MMNs of musicians reflect metrical differences in stimuli better than those of non-musicians^9, 23^. Thus, compared to non-musicians, we expect a stronger effect of meter on subcortical processing in musicians. Further, previous FFR research on musicians found a selective enhancement of behaviorally important features of sounds: for the speech sound /da/, the formant-related components were pronounced in the measured FFR amplitudes^24^. Accordingly, we also expect the formant frequencies to be selectively enhanced when they are presented at metrically higher positions.

## Results

### Metrical modulation of brainstem response to speech

**Figure 1:**
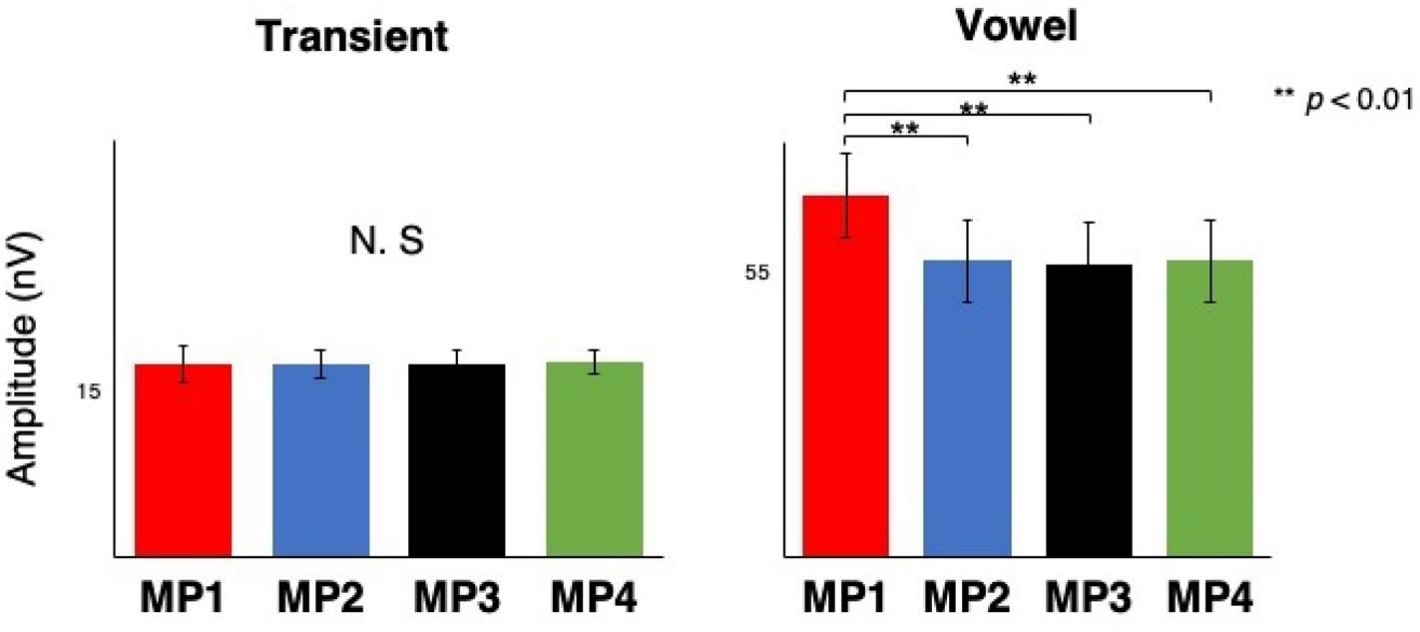
Global spectral subcortical representation of the sound [da] averaged across all frequencies. The strong beat (MP1) showed the highest amplitude in the vowel part. **p < 0.01; error bars represent ± 1 standard error.

**Figure 2:**
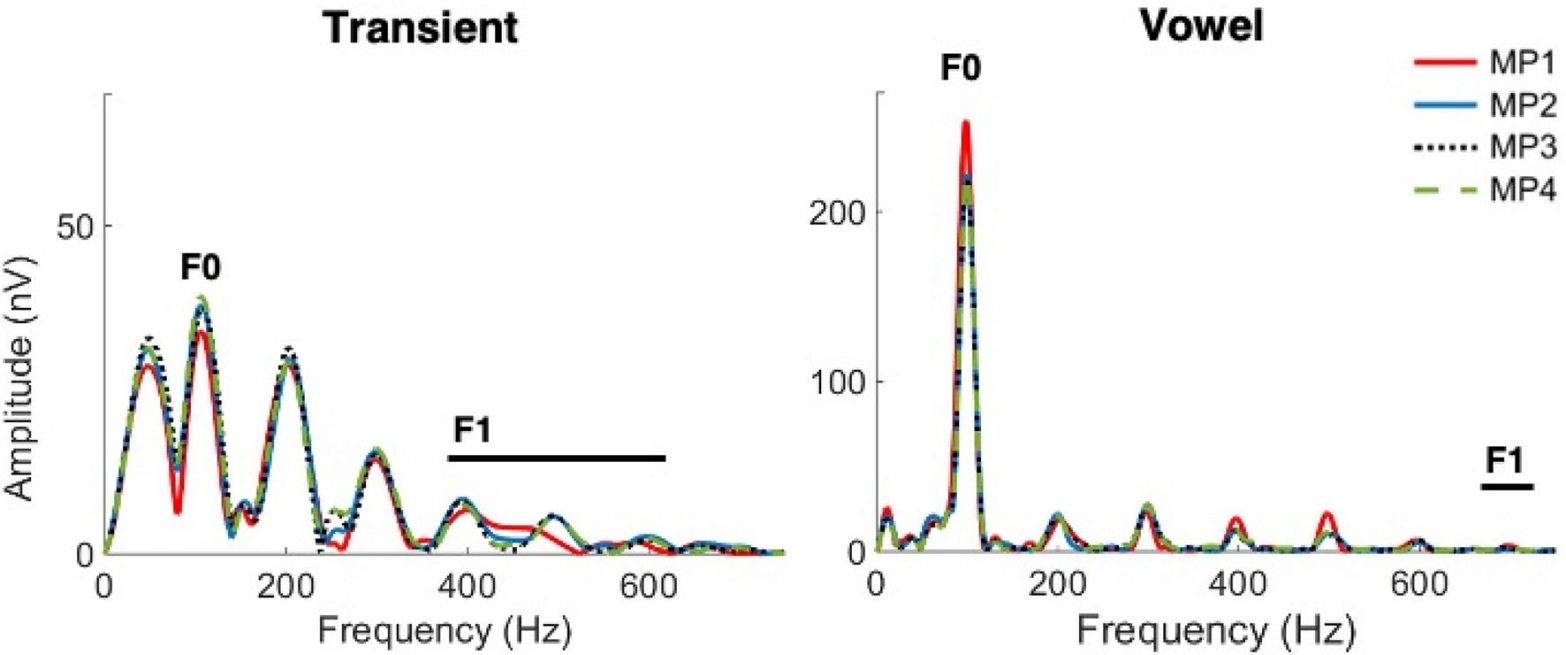
Fast Fourier transform of the neural response to the formant transition (10–60 ms) (left) and the vowel (60–180 ms) (right) at four different metrical positions. The average of all 30 participants is plotted.

Via fast Fourier transform of the neural response to the speech sound (see Methods for details), we first analyzed the effect of meter on the global spectral representation averaged across all spectral components (Fig. 1). Then, to gain a more detailed insight about the effect of meter, we focused on the fundamental (F0) and first formant (F1) frequencies (Fig. 2), which are behaviorally relevant acoustic factors found to show the effect of context in previous studies^14, 16, 17^.

### Global spectral representation

A mixed 2 (time region: transient vs. vowel) × 4 (metrical position: MP1, MP2, MP3, MP4) × (group: musicians vs. non-musicians) repeated-measures analysis of variance (ANOVA) revealed a significant main effect of metrical position (F(2.333, 65.327) = 12.066, *p* = 0.002). The effect of the time region was also significant (F(1, 28) = 165.369, *p* = 0.000). More importantly, there was a significant interaction between time regions and metrical positions (F(1.793, 50.204) = 12.544, *p* = 0.000069), which indicates the different effect of metrical position for each time region. The strong beat, MP1, showed an overall strong neural representation of frequency components only for the vowel part (with Bonferroni correction, *p* = 0.000059 for MP2, *p* = 0.000012 for MP3, *p* = 000059 for MP4) (Fig. 1). The effect of group was not significant.

### Fundamental frequency (F0)

**Figure 3:**
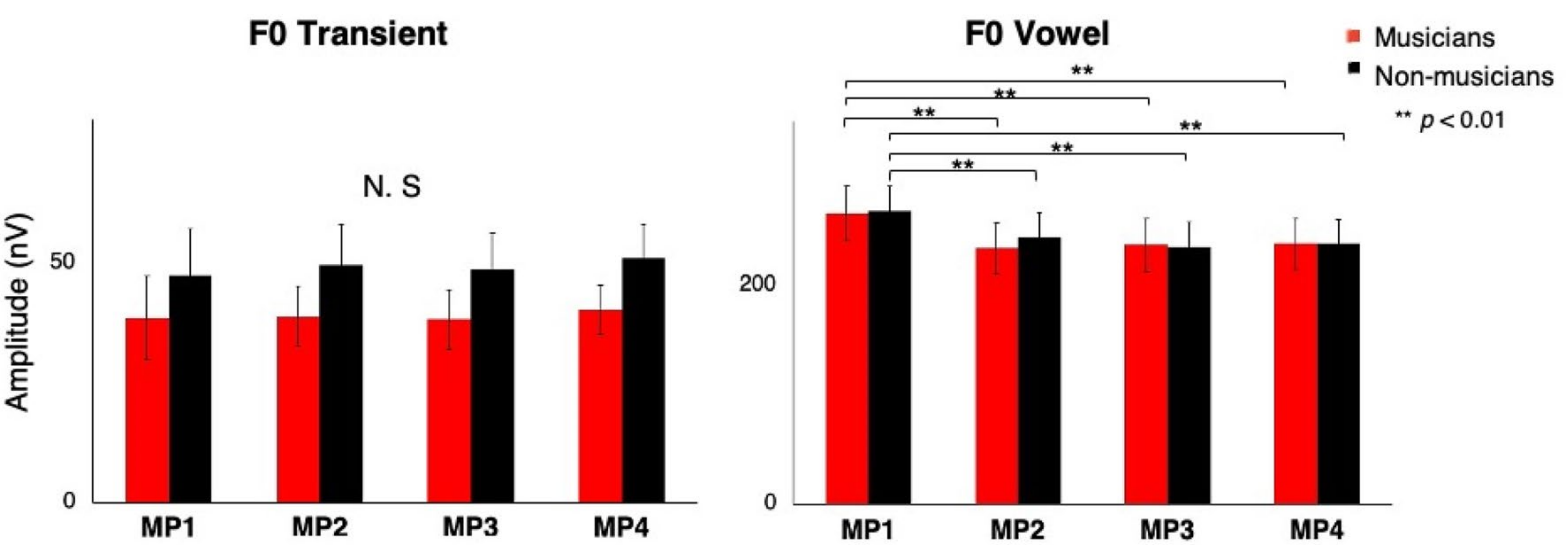
Mean spectral amplitude of the fundamental frequency (F0) for the transient part (left) and the vowel (right). Only the vowel showed significant difference by metrical position. Error bars represent ± 1 standard error.

The strong beat (MP1) showed the highest amplitude in the vowel period, but not in the transient period. The same mixed 2 × 4 × 2 repeated-measures ANOVA as in the previous section revealed a significant effect of metrical position (F(1.884, 52.744) = 19.838, *p* = 0.000). The effect of the time region was also significant (F(1, 28) = 153.795, *p* = 0.000). Interestingly, the interaction between the metrical position and the time region was significant (F(1.743, 48.794) = 29.363, *p* = 0.000). Only the vowel part showed the highest amplitude at MP1 (with Bonferroni correction, *p* = 0.000003 for MP2, *p* = 0.0000 for MP3, *p* = 0.000001 for MP4) (Fig. 3). The group effect was not significant.

### Formant frequency (F1)

**Figure 4:**
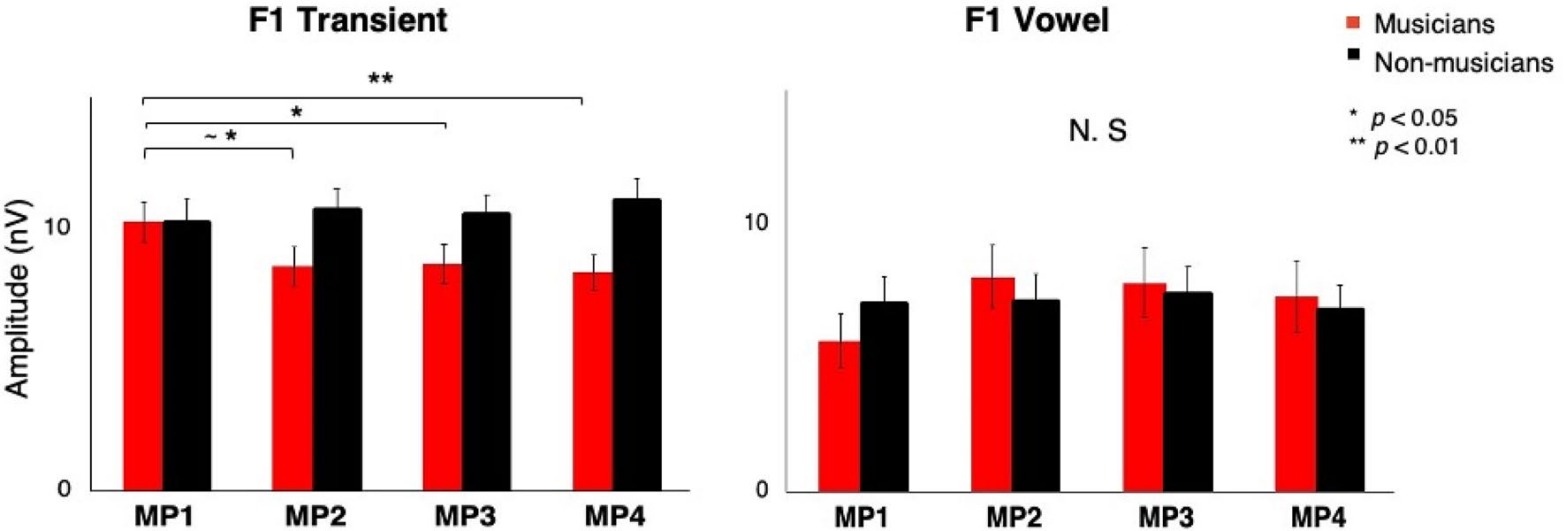
Mean spectral amplitudes of the formant frequency (F1) for the transient part (left) and the vowel (right). For the transient part, only musicians showed the highest amplitude for MP1. Error bars represent ± 1 SE.

For the transient part, only musicians showed the significant effect of metrical position on the formant frequency (400–600 Hz) with the highest amplitude at MP1. For the vowel part, the amplitude of the formant frequency (700 Hz) was not significantly different between the four metrical positions or between groups. A mixed 2 (time region: transient vs. vowel) × 4 (metrical position: MP1, MP2, MP3, MP4) × 2 (group: musicians vs. non-musicians) repeated-measures ANOVA showed a significant effect of time region (F(1, 28) = 12.136, *p* = 0.002). The interaction between time regions and metrical positions was almost significant (F(2.250, 63.012) = 2.694, *p* = 0.069). Most importantly, the interaction between time regions, metrical positions, and groups was significant (F(2.250, 63.012) = 4.006, *p* = 0.019) (Fig. 4).

### Neural consistency

**Figure 5:**
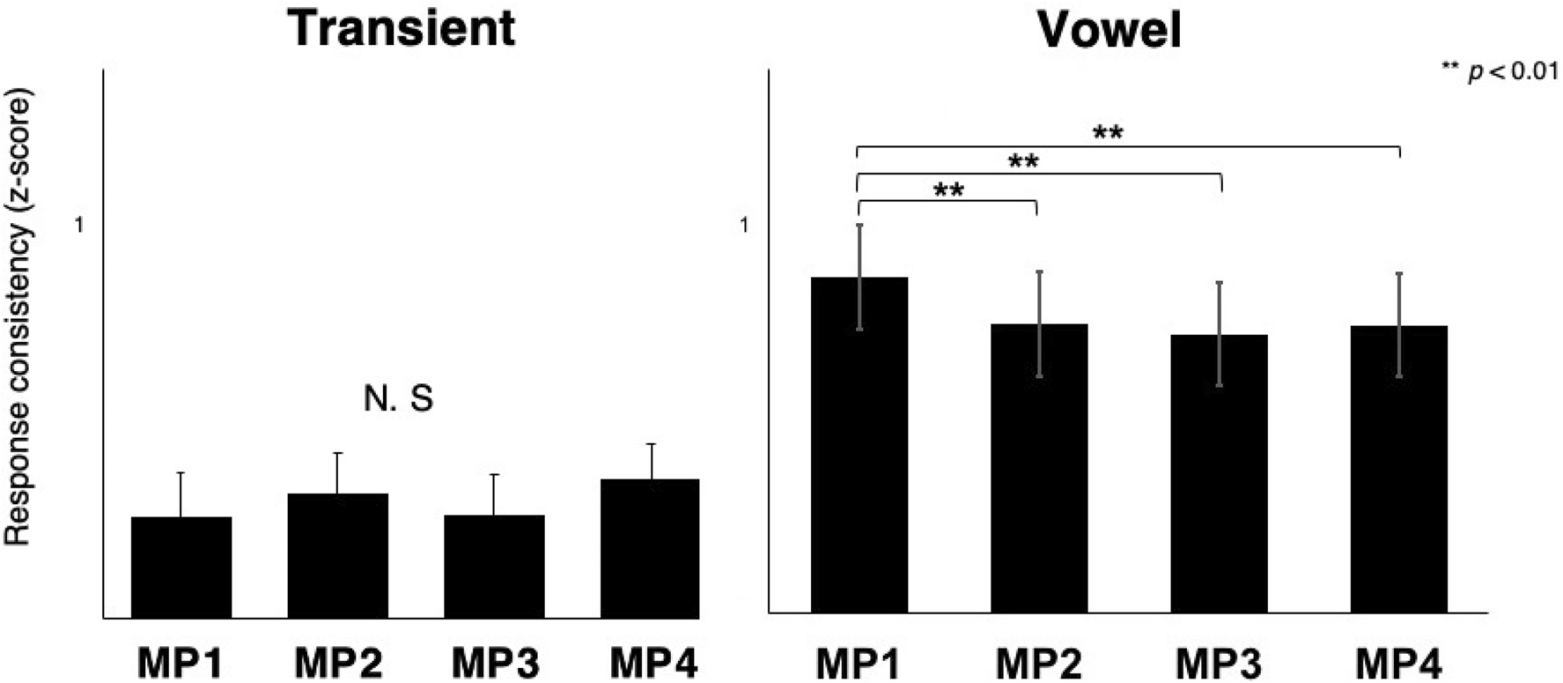
Neural consistency of FFR to the transient (left) and the vowel (right) at four different metrical positions. Only the vowel showed significant difference by metrical position. Error bars represent ± 1 SE.

We assessed trial-by-trial FFR consistency by calculating the correlation between randomly selected pairs of average waveforms. Specifically, for each subject, we first randomly selected 2000 trials among the total 4000 trials and made an average waveform, and then averaged the remaining 2000 trials to make a second average waveform; cross-correlation between the two average waveforms indicated a similarity of response. By repeating this procedure 300 times and averaging the 300 correlation values, we generated a final neural consistency value for individual participants. Neural consistency data were analyzed with a mixed 2 (time region: transient vs. vowel) × 4 (metrical position: MP1, MP2, MP3, MP4) × 2 (group: musicians vs. non-musicians) repeated-measures ANOVA. The effect of time regions was significant (F(1,28) = 30.842, *p* = 0.0006). Further, the interaction between the time regions and metrical positions was significant (F(2.193, 61.412) = 9.493, *p* = 0.000018), which indicates that the effect of metrical position was different for the two time regions. Only the vowel part showed a significant effect of metrical position [F(3, 84) = 9.635, *p* < 0.0001], as shown in Fig. 5, with the highest consistency for MP1 (Bonferroni corrected *p* = 0.000 for MP2, *p* = 0.000 for MP3, *p* = 0.0002 for MP4). The effect of group here was not significant.

## Discussion

To investigate the effect of metrical structure on the encoding of sounds in the brainstem, we measured subcortical electrophysiological responses to speech sounds presented at four metrically different positions. The results showed a significant effect of metrical hierarchy. For the highest metrical position, MP1, the fundamental frequency (F0) of the vowel part of the sound was enhanced; this frequency component is important for the representation of voice pitch. Consistency of the neural responses to the vowel part was also the highest for the highest metrical position. Such results indicate that auditory brainstem responses are modulated by the metrical structure of incoming sounds, which is consistent with prior studies showing that brainstem responses are sensitive to the context of a stimulus. Previous studies found that subcortical responses to sounds presented in highly predictable contexts are more enhanced than those in unpredictable contexts^14–16^. In a musical context, Tierney et al.^25^ found that the alignment of a sound stimulus with the beat of music, as compared to when the sound was shifted away from the beat, enhanced the subcortical response to the sound. In our study, while all stimuli were similarly aligned with the musical beat, here they had different metrical positions, and only the sound at the highest metrical position was found to be subcortically enhanced. We note that previous event-related potential studies have demonstrated that metrical structure modulates early auditory processing; specifically, more negative N1 potentials were found for metrically strong positions compared to metrically weak ones^13^. To our knowledge, the current study is the first to show that the metrical modulation of neural responses extends to the subcortical level.

The difference between musicians and non-musicians was the most significant for the formant frequency of the transient part. At the strong beat, musicians selectively enhanced the acoustic component contributing to formant perception and phoneme discrimination. Previous research has shown that musicians selectively enhance behaviorally relevant acoustic information, such as the upper tone harmonics of a two-tone musical interval^26^ and speech in noise^27^, in their subcortical response. Intartaglia et al.^24^ found that musicians exhibited enhanced subcortical processing of the formant frequencies in foreign languages. Our study demonstrates that musicians’ selective enhancement of formant frequency occurs on the metrically strong beat. Whether the enhancement of F1 in musicians is attributed to their innate neural mechanisms or to their nurtured musical training should be examined with further studies with random assignment to a music intervention.

The enhancement of FFR on strong beats may reflect neural fine-tuning on the strong beat mediated by top-down modulation via the efferent corticofugal network connecting the cortex and lower structures^28, 29^. By associating learned representation and the neural encoding of the physical acoustic features, the corticofugal network has been known to fine-tune subcortical sensory receptive fields of behaviorally relevant sounds in the animal model^30^. With the representation of meter including temporal predictions, the cortex could issue instructions about when to increase the gain to the subcortical regions through top-down feedback. Bolger et al.^31^ found that the functional connectivity of different brain regions peaks at strong beats. Thus, corticofugal modulation would be the most robust at the strong beat, with the peaks of connectivity of the efferent corticofugal network between the cortical and subcortical levels. As musicians have a stronger representation of meter, more fine-tuned subcortical processing is available to them at the strong beat.

Alternatively, it may be possible that subcortical enhancement at the strong beat is attributable to its acoustic saliency, since the sound of the strong beat was provided with a deviant higher pitch having a lower probability (25%). With this interpretation, automatic attention driven primarily by acoustic saliency could enhance subcortical processing at the strong beat. However, it has been found that the deviancy or novelty of a stimulus reduces the spectral amplitude of the higher harmonics in the brainstem response^19^. In addition, it has been found that musicians are more sensitive to stimulus probability, showing reduced brainstem responses to a speech sound when it is presented infrequently compared to when it is repeated^16^. The musicians in our study, though, showed enhanced amplitudes for the infrequent stimulus, and thus, it is more probable that the subcortical enhancement observed in this study is the effect of metrical structure. To further investigate whether the amplitude enhancement for the strong beat is the effect of metrical structure or acoustic saliency, we plan to execute additional experiments with metrical structure using rhythmic patterns without changes in pitch, loudness, or timbre. We expect such future studies to clarify the effect that metrical structure has on subcortical processing.

In this study, the strong beat was implied by the high pitch. While it has been known that note duration is the best predictor of meter^32^, Hannon et al.^33^ demonstrated that melodic accents as well as temporal features predict listeners’ meter perception. Although they did not provide direct evidence supporting the role of high pitch in meter perception, they found contour change and melodic repetition are important factors predicting meter judgement. In our study, a sound pattern composed of A7 (3520 Hz), A6 (1760 Hz), A6 (1760 Hz), and A6 (1760 Hz) was repeated. Here in this stimulus, we intended to mark the beginning of the repeating pattern by using the change of the melodic contour and the octave leap that the high pitch (A7) of the strong beat makes. Leardahl and Jackendoff^34^ also suggested the possibility of phenomenal accent made with the interval leap. Further, compared to leaps to low pitch, leaps to relatively high pitch make more stress^35^. It is therefore possible that the phenomenal accent made by the leap to high pitch in our stimulus contributed to the perception of the quadruple meter. Future studies with more clear-cut indicators of metrical strength, such as repeating temporal patterns, could provide clearer evidence supporting the contribution of meter on the subcortical processing.

Scalp-recorded FFR has long been thought to reflect subcortical auditory activity. In fact, the FFR directly recorded from subcortical structures is remarkably similar to scalp-recorded FFR^36^. Lesion studies also found that patients with brainstem lesions or subcortical neuropathy showed no FFR, whereas those with bilateral auditory cortex lesions showed robust FFR^37, 38^. Source modeling based on FFR recorded with high-density, multichannel EEG also demonstrated that FFR reflects auditory subcortical activity^39, 40^. In contrast, it has recently been suggested that the FFR is an aggregate measure reflecting a mixture of sources including the brainstem, midbrain, thalamus, and auditory cortex^41, 42^. Indeed, magnetoencephalography (MEG) evidence demonstrates the cortical contribution to FFR^43^, and a study with a combination of EEG and functional magnetic resonance imaging (fMRI) also indicated the contribution of the auditor cortex to FFR by showing the relation between the fMRI response in the right auditory cortex and the EEG-based FFR response^44^. However, the contribution of each source could differ depending on where and how the response is recorded^42^. By using a vertical montage with an earlobe reference, the current study minimized the contribution of peripheral sources^45, 46^. Given the upper frequency limit of the auditory cortex for phase-locking, cortical contribution could also be excluded. The FFR reflects the response generated by the phase-locking of neuronal ensembles in the auditory pathway. Since the auditory cortex phase-locks only up to about 100 Hz^47^, it is not likely that frequency components higher than 100 Hz in the FFR are generated by the auditory cortex. In our results, the F1 of the transient part was 400–600 Hz, so we can clearly know this is not related to cortical activities. However, it remains to be examined whether the enhanced F0 (100 Hz) of the periodic part is really the effect of meter on the subcortical level. Further studies using the MEG-FFR approach could disentangle the cortical contribution from the response.

In summary, we found that the FFR amplitude of the F0 and F1 as well as neural consistency were enhanced at the metrically strongest beat, indicating that meter has modulatory effects on the subcortical processing of sound. Compared to non-musicians, musicians showed heightened FFR amplitudes on the strong beat, especially for the behaviorally relevant acoustic component, i.e. the formant frequency, demonstrating a stronger effect of meter in a way that the selective enhancement of sound is facilitated on the strong beat. Taken together, the findings of this study suggest that metrically strong beats are processed differently at the brainstem level.

## Materials and methods

### Participants

Thirty adults ranging in age from 19 to 27 years (mean age 22.73 years) participated in this study. Subjects completed a questionnaire that assessed musical experience in terms of beginning age, length, and type of performance experience. Fifteen (all females, mean age 22 years) were musicians (12 pianists, 2 violinists, and 1 violist) with 10 or more years of musical training that began at or before the age of 7. Fifteen (12 females and 3 males, mean age 24.2 years) were non-musicians with < 3 years of musical training. All participants reported no audiologic or neurologic deficits and had pure tone air conduction thresholds < 20 dB HL for octaves from 125 to 8000 Hz. This study was approved by the Samsung Medical Center Institutional Review Board (SMC2017-01-115-016) and was in accordance with the Code of Ethics of the World Medical Association (Declaration of Helsinki). Written informed consent was obtained from each participant before starting the experiment.

### Stimulus

The stimulus was a 170 ms six-formant stop consonant vowel speech syllable, [da], synthesized using a Klatt-based formant synthesizer at a 20 kHz sampling rate (for more information on the syllable, see Parbery-Clark et al.^27^). The syllable was composed of a 50 ms formant transition and a 120 ms steady-state vowel. The fundamental frequency of the syllable (F0 = 100 Hz) was steady throughout the stimulus. The first, second, and third formants changed over time for the first 50 ms (*F*_1_: 400 to 720 Hz, *F*_2_: 1700 to 1240 Hz, *F*_3_: 2580 to 2500 Hz), while being consistent for the subsequent 120 ms. The fourth, fifth, and sixth formants did not change throughout the stimulus (*F*_4_ = 3300 Hz, *F*_5_ = 3750 Hz, *F*_6_ = 4900 Hz). The interstimulus interval was 500 ms. To prime a quadruple meter such as 4/4, the syllable [da] was presented with a repeating series of four tones: 3520 Hz (A7), 1760 Hz (A6), 1760 Hz (A6), and 1760 Hz (A6). The frequencies of the four tones did not overlap with the frequency components of the syllable. The duration of each tone was 100 ms.

### Electrophysiological recordings

The stimulus was binaurally presented via inset earphone (ER-3A) at an intensity (sound pressure level) of approximately 65 dB (Neuroscan Stim2; Compumedics) with alternating polarities to eliminate the cochlear microphonic response. During testing, subjects watched a muted movie of their choice with subtitles. Data collection followed procedures outlined in Lee et al.^26^. Brain responses were collected using Scan 4.3 Acquire (Neuroscan; Compumedics) with four Ag–AgCl scalp electrodes, differently recorded from Cz (active) to linked earlobe references with forehead ground. Contact impedance was < 5 kΩ for all electrodes. About 2000 sweeps were collected at each stimulus polarity with a sampling rate of 20 kHz.

### Data analysis

Filtering, artifact rejection, and averaging were performed off-line using Scan 4.3 (Neuroscan; Compumedics). To isolate the contribution of the brainstem, responses were bandpass filtered from 70 to 2000 Hz (12 dB/oct roll off), and trials with activity greater than ± 35 μV were rejected as artifacts, such that 2000 remaining sweeps were averaged. Responses of alternating polarities were added together to isolate the neural response by minimizing the stimulus artifact and cochlear microphonic^48^. The responses were divided into two time ranges: the formant transition of the stimulus (10–60 ms in the neural response) and the steady-state vowel (60–180 ms). Over the transition part, spectral magnitudes were calculated for 20 Hz bins surrounding the F0 and subsequent six harmonics (100 Hz [F0], 200 Hz [H2], 300 Hz [H3], 400 Hz [H4], 500 Hz [H5], 600 Hz [H6], and 700 Hz [H7]), while 10 Hz bins were used over the vowel.

Following the procedure previously used in Hornickel and Kraus^49^, trial-by-trial FFR consistency was assessed by calculating the correlation between randomly selected pairs of average waveforms. Average waveforms were created by averaging 2000 randomly selected trials and the remaining 2000 trials, where cross-correlation of the two average waveforms can indicate the similarity of response. Final neural consistency values for all individual participants were then generated by repeating this procedure 300 times and averaging the 300 correlation values. Response consistency data were Fisher transformed.

### Statistics

Statistical analyses were performed in SPSS. A mixed 2 (time region: transient vs. vowel) × 4 (metrical position: MP1, MP2, MP3, MP4) × 2 (group: musicians vs. non-musicians) repeated-measures ANOVA was used for spectral and consistency analysis. Where appropriate, the Greenhouse–Geisser correction was applied in the ANOVAs.

### Conference presentation

The part of results was presented as an oral presentation at the 2019 biennial meeting of the Society for Music Perception and Cognition, New York, August 2019.
